# Influence of Actinidin-Induced Hydrolysis on the Functional Properties of Milk Protein and Whey Protein Concentrates

**DOI:** 10.3390/foods12203806

**Published:** 2023-10-17

**Authors:** Surjit Kaur, Todor Vasiljevic, Thom Huppertz

**Affiliations:** 1 Advanced Food Systems Research Unit, Institute for Sustainable Industries & Liveable Cities, College of Health and Biomedicine, Victoria University, Melbourne, VIC 8001, Australia; surjit.kaur@live.vu.edu.au (S.K.); todor.vasiljevic@vu.edu.au (T.V.); 2 FrieslandCampina, 3818 LE Amersfoort, The Netherlands; 3 Food Quality and Design Group, Wageningen University & Research, 6708 WG Wageningen, The Netherlands

**Keywords:** actinidin, proteolysis, milk protein concentrate, whey protein concentrate, functional properties

## Abstract

The main aim of the study was to establish the impact of limited proteolysis by actinidin on the functionality of selected milk protein systems. The plant protease actinidin was used to produce hydrolysates (MPHs) from milk protein concentrate (MPC) and whey protein concentrate (WPC) to 0, 5, 10 or 15% of the degree of hydrolysis (DH) at an enzyme-to-substrate ratio of 1:100 (5.21 units of actinidin activity g^−1^ of protein). The functionalities assessed included solubility, heat stability, emulsification and foaming properties. In general, significant changes in the functionalities of MPH were associated with the extent of hydrolysis. Solubility of hydrolysates increased with increasing %DH, with WPC showing about 97% solubility at 15% DH. Emulsifying properties were negatively affected by hydrolysis, whereas heat stability was improved in the case of WPC (~25% of heat stability increased with an increase in DH to 15%). Hydrolysates from both WPC and MPC had improved foaming properties in comparison to unhydrolysed controls. These results were also supported by changes in the FTIR spectra. Further adjustment of hydrolysis parameters, processing conditions and pH control could be a promising approach to manipulate selected functionalities of MPHs obtained using actinidin.

## 1. Introduction

Milk protein ingredients, including milk protein concentrate (MPC) and whey protein concentrate (WPC), are frequently used in nutritional and cultured dairy products and for protein standardisation and production of processed cheeses [1,2,3]. However, some of their applications in food systems are hindered by functionality issues. For example, high viscosity or poor solubility (at room temperature and neutral pH) leads to limitations with the utilisation of these proteins in high-energy drinks [3,4]. Furthermore, the emulsification and foaming properties of MPC are poorer than those of whey proteins (WPs), which can limit its usage in processed meats, soups, coffee creamers and whipped toppings [4,5]. Prolonged storage and elevated storage temperatures of milk protein powders such as MPC85 (containing 85% of proteins on dry matter) may lead to a rise in insolubility due to protein-protein interactions as a result of the creation of junction zones among adjacent protein powder particles [6]. Heat-induced destabilisation, especially of whey proteins, may cause phase separation or protein precipitation in the final products, such as in heat-treated beverage drinks [7].

Modifying functional properties, such as solubility, viscosity, emulsification and foaming, by enzymatic hydrolysis is one of the approaches to improve some of the properties of these proteins [8]. A study conducted by Ryan et al. [8] showed that protein hydrolysis rate resulted in greater solubility and reduced viscosity of milk protein isolate (MPI), which was attributed to a change in the protein structure, size and hydrophobicity of the released peptides. Damodaran [9] suggested that many factors affect the foaming properties of proteins, such as the type of enzyme used, temperature, protein conformation and concentration, pH, mixing time, speed of whipping and foaming method. Furthermore, Banach et al. [2] also showed an improvement in nitrogen solubility of MPC80 hydrolysates after trypsin, pepsin, chymotrypsin or papain hydrolysis.

Current knowledge shows that changes in the functionality of milk protein hydrolysates depend on proteases used in their creation. While enzymes of animal and microbial origins have been used in the production and hydrolysis of dairy products, for example, chymosin, more attention has been placed more recently on plant-based proteases due to their availability and feasibility of extraction, especially from plant waste streams such as peels and rejects. Actinidin (EC 3.4.22.14), a plant-based cysteine protease (CA1) with a molecular weight of 23.5 kDa, is extracted from kiwi fruit. Actinidin can act in a wide range of temperatures (15–30 °C) and pH (4–10) with a broad substrate specificity [10]. The enzyme was recently assessed for potency to alleviate the antigenicity of two proteins in MPC and WPC, β-lactoglobulin and α_s1_-casein [11]. The extent of antigenicity reduction was clearly dependent on the degree of hydrolysis. Furthermore, milk proteins appeared to be only partially hydrolysed by actinidin [10]. This limited hydrolysis clearly changes the conformation of the proteins in these preparations, which consequently indicates that their functionality may be affected as well [10,12,13]. For example, research has been conducted on the hydrolysis of milk protein concentrates with papain, an enzyme with a similar specificity to actinidin, which resulted in improved solubility at pH 7 [2]. Also, Al-Shamsi, Mudgil, Hassan, & Maqsood [14] showed substantially improved emulsification expressed as emulsifying activity index (EAI) when camel milk proteins were hydrolysed with papain as compared to that of the control. On the other hand, bromelain, a protease from pineapple, had no impact on EAI when used on the same substrate.

Therefore, the present study was carried out to establish whether milk protein hydrolysis by actinidin would have an impact on selected functional properties of MPC and WPC. The focus was on solubility, heat stability, foaming and emulsification properties, especially since the latter two are also related to solubility [15].

## 2. Materials and Methods

### 2.1. The Materials

WPC (80%, *w*/*w*, protein on dry matter) and MPC (80%, *w*/*w*, protein on dry matter) were obtained from Fonterra Cooperative (Palmerston North, New Zealand). Actinidin (KEP500 with 521 activity units g^−1^) was kindly provided by kiwiEnzyme.com Ltd. (Martinborough, New Zealand). Trinitrobenzenesulfonic acid (TNBS), sodium phosphate buffer (0.2125 M, pH 8.2) and sodium dodecyl sulphate (SDS) were of analytical grade and were obtained from Sigma-Aldrich Pvt Ltd. (Castle Hill, NSW, Australia) and Merck KGa (Darmstadt, Germany). Simulated milk ultrafiltrate (SMUF, pH 7) [16] was used as a buffer during hydrolysis [17].

### 2.2. Sample Preparation and Enzymatic Hydrolysis of Milk Protein Systems

**Figure 1:**
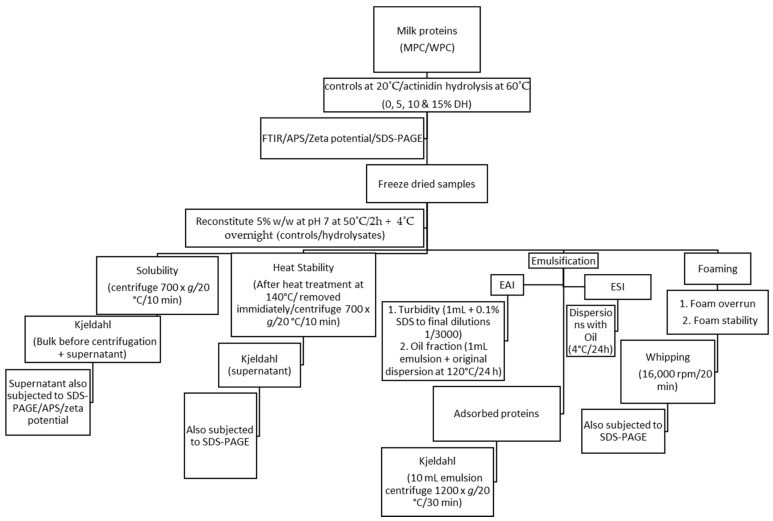
Experimental design used in the study. MPC = of milk protein concentrate; WPC = whey protein concentrate; FTIR = Fourier transform infrared spectroscopy; SDS-PAGE = Sodium Dodecyl Sulphate Polyacrylamide Gel Electrophoresis; APS = Average particle size; EAI = Emulsifying activity index; ESI = emulsion stability index.

Protein dispersions (5%, *w*/*w*) were prepared by dispersing WPC or MPC in SMUF as described previously [10]. The control samples (0% DH) were prepared at room temperature (20 °C) in SMUF without the addition of the enzyme. Actinidin was added at the enzyme to substrate ratio (E:S) of 1:100 (5.21 units of actinidin activity per g of protein), and subsequently, each trial was performed at 60 °C until the 5, 10 or 15% degrees of hydrolysis (DH) was achieved. The %DH was assessed by the trinitrobenzenesulfonic acid (TNBS) procedure, as described previously [10]. The experimental design applied in the current study is depicted in Figure 1. Total protein was determined using a Kjeldahl method with a nitrogen conversion factor of 6.38 [18]. For functional properties, the controls were prepared under the same conditions (at 50 °C) but without enzymatic treatment. Hydrolysates were heat treated at 85 °C for 10 min without adding SDS to inactivate the enzyme [19,20]. The samples were then freeze-dried using a pilot-scale freeze dryer (model FD-300, Airvac Engineering Pty. Ltd., Dandenong, Australia), followed by storing them in plastic airtight containers at ambient temperature for further analysis. For SDS-PAGE analysis, exactly 50 µL of the sample was preserved in 950 µL of SDS sample buffer and then stored at −20 °C for further testing.

### 2.3. Particle Size and Zeta Potential Measurement

Straight after hydrolysis, average particle size (APS) and zeta potential (ζ-potential) of all the controls and hydrolysed samples were determined by a Zetasizer-Nano ZS (Malvern Instruments, Malvern, UK) [21].

### 2.4. Sodium Dodecyl Sulphate Polyacrylamide Gel Electrophoresis (SDS-PAGE)

The obtained hydrolysates were analysed by SDS-PAGE to study individual milk protein during hydrolysis. The analysis was performed under non-reducing and reducing (using β-mercaptoethanol) conditions as described previously [11]. Gels were scanned with the ChemiDoc imager (Chemidoc MP, Bio-Rad Laboratories, Hercules, CA, USA), and gels quantifications were performed for all reducing gels of both substrates in triplicate with software Image Lab 6.0.1 @2017, Bio-Rad Laboratories Inc.

### 2.5. Fourier Transform Infrared Spectroscopy (FTIR)

Immediately after treatment, FTIR spectra were obtained using a PerkinElmer Frontier FTIR spectrometer (PerkinElmer, Boston, MA, USA), as stated in our previous work [10]. Following peak areas were identified with four peak areas closely examined, including side chains (1607–1602 cm^−1^), β-sheets (1640–1608 cm^−1^ and 1693–1680 cm^−1^), random coils (1648–1642 cm^−1^), α-helices (1663–1649 cm^−1^), and β-turns (1678–1666 cm^−1^) [22].

### 2.6. Functional Properties of MPs

The functional properties of freeze-dried powders were analysed by preparing 5% *w*/*w* (protein base) of the protein dispersions (controls and hydrolysed samples) at 50 °C for approximately 2 h under constant stirring, followed by overnight storage at 4 °C to allow for full hydration. The final weight was corrected with a pH adjustment to 7 using 1 M NaOH or 0.1 M HCl.

#### 2.6.1. Determination of Protein Solubility

Each dispersion after hydrolysis of resuspended material after freeze-drying was centrifuged (Model J2HS; Beckman, Fullerton, CA, USA) at 700× *g* for 10 min at 20 °C [1], and their supernatants were collected. The protein content of their original dispersions and the resultant supernatants were then quantified by the Kjeldahl method using 6.38 as a conversion factor for both WPC and MPC [18], and protein solubility was expressed using the equation below [23].

These supernatants were also analysed by SDS-PAGE, and their APS and zeta potential were measured as described above.

(1) $$\%\;\mathrm{Solubility}\;=\frac{\mathrm{protein}\;\mathrm{content}\;\mathrm{of}\mathrm{supernatant}\;(\mathrm{mg}\;{mL}^{-1})\;}{\mathrm{protein}\;\mathrm{content}\;\mathrm{of}\;\mathrm{corresponding}\;\mathrm{dispersion}\;(\mathrm{mg}\;{mL}^{-1})\;}\cdot 100$$

#### 2.6.2. Determination of Heat Stability

The heat stability of protein dispersions was examined by establishing the solubility of the dispersions after exposure to a high temperature. A protocol described by Dissanayake et al. [23] was followed with samples treated in an oil bath at 140 °C. The time once samples reached 140 °C was recorded (WPC for 2.1 min; MPC for 2.66 min), and samples were immediately removed from a Riotek oil bath, followed by instant cooling in an ice slurry and centrifuged at 700× *g* for 10 min at 20 °C (Model J2HS). The protein quantification of supernatants of heated and original samples was conducted by the Kjeldahl method as per Section 2.6.1 using 6.38 as a conversion factor for both WPC and MPC (method 968.06) [18]. Heat stability was expressed using the equation below [23].

(2) $$\%\;\mathrm{Heat}\;\mathrm{stability}\;=\frac{\mathrm{protein}\;\mathrm{content}\;\mathrm{in}\;\mathrm{supernatant}\;\mathrm{after}\;\mathrm{heating}\;(\mathrm{mg}\;{mL}^{-1})}{\mathrm{protein}\;\mathrm{content}\;\mathrm{of}\;\mathrm{corresponding}\mathrm{supernatant}\;\mathrm{prior}\;\mathrm{to}\;\mathrm{heating}\;(\mathrm{mg}\;{mL}^{-1})\;}\cdot 100\;$$

#### 2.6.3. Determination of Emulsifying Properties

Emulsifying activity index (EAI), emulsion stability index (ESI) and protein adsorption of each sample were analysed by a turbidimetric technique described by Cameron, Weber, Idziak, Neufeld, & Cooper [24] and modified by Dissanayake et al. [23]. The EAI of samples were calculated using the following equation expressed as units of area of interface stabilised per unit weight of protein:

(3) $$EAI=\frac{2\;\cdot \;T\;}{\left(1-\Phi \right)\;\cdot \;C}$$

where T denotes turbidity, Φ is the oil volume fraction, and C is the weight of protein per unit volume of aqueous phase before an emulsion is formed.

ESI was estimated after holding the emulsions at 4 °C for 24 h using the following formula:

(4) $$ESI=\frac{(T\;\cdot \;\Delta t)\;}{\;\Delta T}$$

where T is the turbidity value at zero h; Δt is the time interval in hrs; ΔT is turbidity after Δt [25].

The amount of adsorbed protein was calculated by the equation:

(5) $$Adsorbed\;protein\;(\mathrm{mg}\;{mL}^{-1})=protein\;in\;stock\;solution\;(\mathrm{mg}\;{mL}^{-1})-protein\;in\;aqueous\;layer\;of\;emulsion\;(\mathrm{mg}\;{mL}^{-1})$$

#### 2.6.4. Determination of Foaming Properties

Foaming properties were determined according to the method described by Phillips et al. [26] with minor modifications stated by Dissanayake et al. [23]. Foam overrun was calculated using the following equation:

(6) $$\mathrm{Overrun}\;(\%)\;=\frac{(\mathrm{wt}.\;\mathrm{of}\;100\;\mathrm{mL}\;\mathrm{sample}\;\mathrm{suspension})\;-\;\left(\mathrm{wt}.\;\mathrm{of}\;100\;\mathrm{mL}\;\mathrm{foam}\right)}{\mathrm{wt}.\;\mathrm{of}\;100\;\mathrm{mL}\;\mathrm{foam}}\cdot 100$$

Foam stability was measured by monitoring the drainage of liquid at ambient temperature, as described by Dissanayake et al. [23]. It was defined as a time to attain 50% drainage of the original weight of the dispersion [26].

### 2.7. Statistical Analysis

The experiments were conducted in a randomised split block design with the extent of hydrolysis as the main factor and repetitions as the block. The design was replicated at least three times on separate occasions for both substrates, and the data were expressed as the mean ± SD of three independent assays. In addition, for the Kjeldahl analysis, the analytical determination was replicated twice, followed by a subsampling (*n* = 4). The data was analysed by two-way ANOVA using the SAS software (v. 9.1). The means were compared using the Tukey multi-comparison, and the significance level was set at *p* < 0.05.

## 3. Results

### 3.1. Changes in Particle Size and Composition of Milk Protein Hydrolysates Obtained by Actinidin-Induced Hydrolysis of Milk Protein Concentrate and Whey Protein Concentrate

As Table 1 indicates, the bulk of the MPC control had an average particle size of 295 nm, which rose with an increase in %DH, reaching 343 nm at 15%DH. At the same time, the average size of the particles in the MPC supernatant was reduced significantly from that of the control (282 nm) down to 171 nm. On the contrary, the average particle size of both bulk and supernatant of the WPC control was 426 and 386 nm and declined to 410 and 353 nm, respectively, with an increase in DH (15%). However, this particle size (as large as fat globules) can be attributed to fat globules size due to the presence of residual lipid content in WPC powder [27].

Also, as indicated by Table 1, the zeta potential of both bulk and supernatants of WPC control was −9 mV. After attaining 15% DH, zeta potential became more negative, reaching −13 and −14 mV for the bulk and supernatants, respectively. Similarly, the MPC underwent a comparable increase in negative zeta potential from −15 mV (0% DH) to −18 mV (15% DH) for the supernatants. However, the MPC bulk had no apparent trend, which could be assigned to the heterogeneity of proteins as opposed to that of WPC; thus, the changes may have been various.

**Figure 2:**
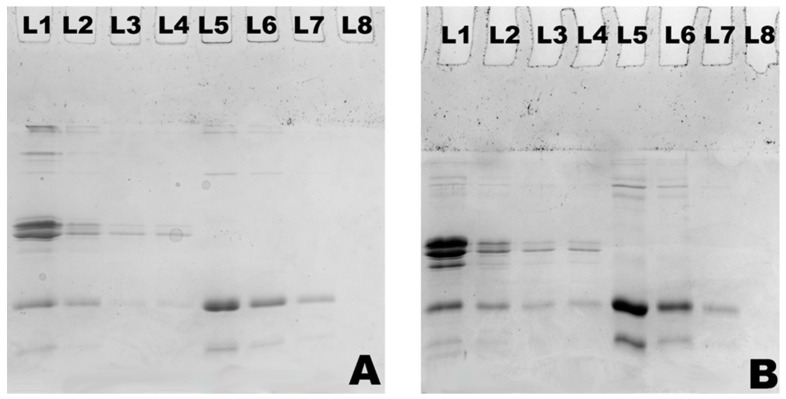
Non-reducing (**A**) and reducing (**B**) SDS-PAGE patterns of hydrolysates of heated supernatants for heat stability of MPC (L1–L4) and WPC (L5–L8) obtained by actinidin treatments at 60 °C with 0 (L1, L5), 5 (L2, L6), 10 (L3, L7) and 15% DH (L4, L8).

**Figure 3:**
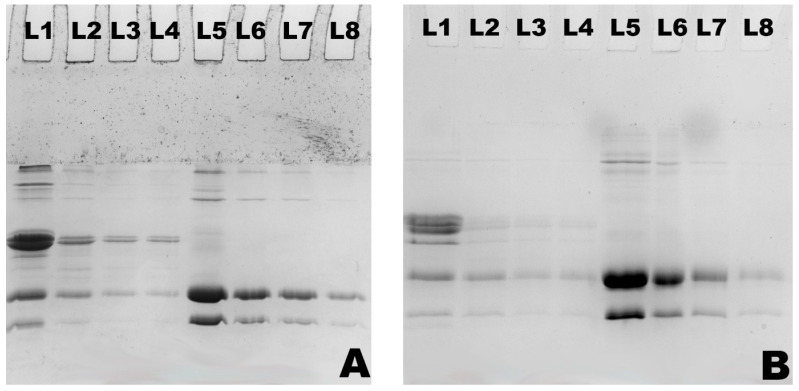
Non-reducing (**A**) and reducing (**B**) SDS-PAGE patterns of MPC (L1–L4) and WPC (L5–L8) hydrolysates of drained liquid for foaming obtained by actinidin treatments at 60 °C with 0 (L1, L5), 5 (L2, L6), 10 (L3, L7) and 15% DH (L4, L8).

**Figure 4:**
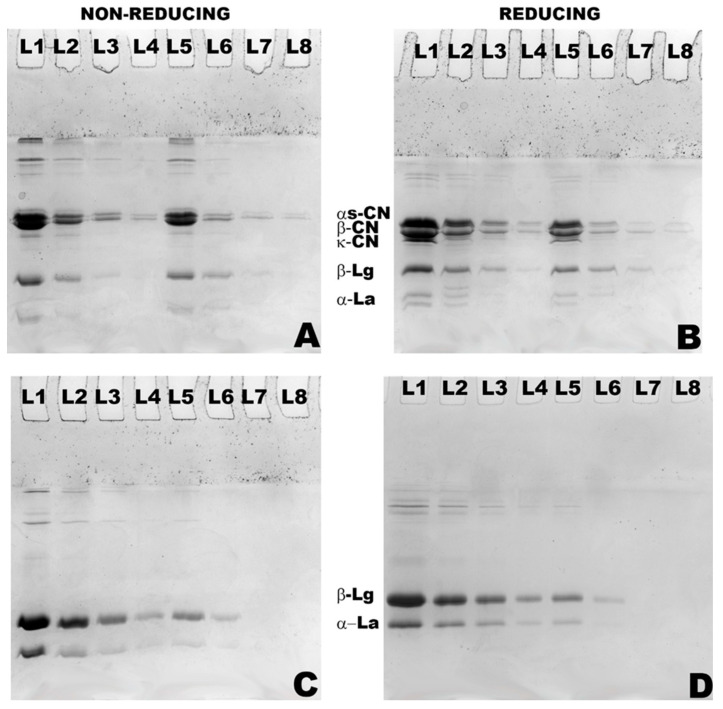
Non-reducing (**A**,**C**) and reducing (**B**,**D**) SDS-PAGE patterns of hydrolysates of original (L1–L4) and supernatants (L5–L8) for solubility of MPC (**A**,**B**) and WPC (**C**,**D**) obtained by actinidin treatments at 60 °C with 0 (L1, L5), 5 (L2, L6), 10 (L3, L7) and 15% DH (L4, L8).

The PAGE patterns of MPC and WPC hydrolysates are shown in Figure 2, Figure 3 and Figure 4, which demonstrate the nature and extent of protein interactions. Protein patterns were compared, and they appear to be in agreement with the %DH and solubility (Figure 2, Figure 3 and Figure 4; Table 2, Table 3 and Table 4). As expected, casein bands (α_S_-, β-, and κ-CN) were detected in the MPC samples only, and those of major whey proteins, β-LG and α-LA, were detected in both MPC and WPC samples. At the highest DH (15%), there was <10% and 5% of each casein remaining after hydrolysis in the case of MPC bulk and supernatant, respectively (Figure 2B; Table 2). In the WPC bulk (Figure 4D; Table 2), ~21% and ~25% of β-LG and α-LA remained, whereas no bands were detected in the WPC supernatants, indicating that released peptides were not retained in the gel.

### 3.2. Modification of Secondary Structure in Milk Protein Hydrolysates Obtained by Actinidin

All peaks assigned to a specific FTIR region were selected carefully, including the otherwise hidden peaks that were only possible to see in spectra in a stacking form of peaks (Supplementary Materials) to determine the main proteins’ structural changes during processing and their interactions. The current study showed an inverse trend in the peak areas for β-sheets and α-helical structures, while negligible changes for random coils and β-turns took place for both MPC and WPC (Table 5). In the case of the MPC, the peak areas assigned to β-sheets significantly increased (*p* < 0.05), approximately by about 6% and 11% in the samples with 10% and 15% DH, respectively, in comparison to that of the control. Simultaneously, the sample with 10% DH demonstrated a substantial reduction of α-helix peak area by ~2%, with a further ~6% decrease in this peak area when the sample was further hydrolysed (15% DH), compared to that of the control. In the case of the WPC, a rise of ~4% in the peak areas associated with β-sheets was observed in both 10–15% DH samples compared to that of the control. At the same time, the α-helix peak area decreased by ~5% at the maximum DH compared to the control. Interestingly, this study showed a clear trend, but contrary to previously reported FTIR data, where only limited changes in the secondary structure of MPC and WPI were observed [11].

### 3.3. Functional Properties of Hydrolysates

#### 3.3.1. Functional Properties of MPC Hydrolysed by Actinidin

**Figure 5:**
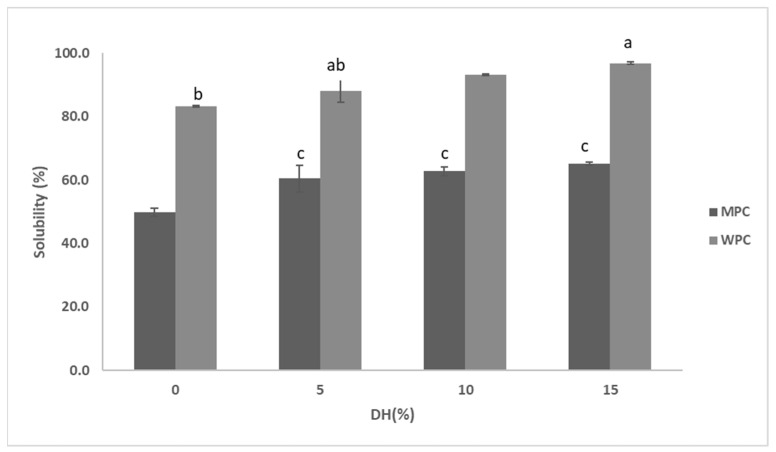
Solubility percentage of hydrolysates of 5% (*w*/*w*) dispersions of MPC and WPC with actinidin at 60 °C at 0, 5, 10 and 15%DH. The values with different lower-case letters indicate significant differences (*p* < 0.05).

**Figure 6:**
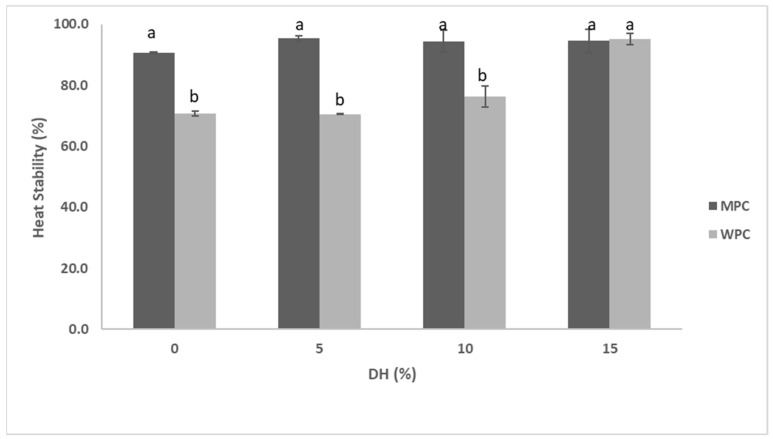
Heat stability % of hydrolysates of 5% (*w*/*w*) dispersions of MPC and WPC with actinidin at 60 °C at 0, 5, 10 and 15% DH. The values with different lower-case letters are significantly different (*p* < 0.05).

Enzymatic hydrolysis affected the solubility of MPC in a % DH-dependent manner. The solubility of the control sample was about 50%, which was improved to ~60% and the most to 65% at 5% and 15% DH, respectively (Figure 5). Heat stability appeared to follow the same pattern—greater solubility led to improved heat stability (Figure 6). However, heat stability was not clearly dependent on the %DH. The control was characterised with 90.7% heat stability, which further improved to 95.4% when MPC was treated to 5% DH. This was basically the maximum heat stability the hydrolysed samples were able to reach as other samples at greater %DH remained at this level (Figure 3 and Table 3). This can also be seen in Figure 3B and Table 3, in which MPC supernatant at 15%DH showed total disappearance of bands taking place only in the case of α-LA and about 10% of αs- and κ-CN remained along with about 20% of β-CN and 26% β-LG.

The emulsifying activity index (EAI) of the MPC samples significantly (*p* < 0.05) decreased with an increase in %DH. The control MPC sample was characterised with the greatest EAI (17.45 m^2^ g^−1^), which decreased to 13.80 m^2^ g^−1^, 11.58 m^2^ g^−1^ and the lowest to 9.49 m^2^ g^−1^ upon hydrolysis to 5%, 10% and 15% DH, respectively (Table 6). Furthermore, the emulsion stability of all samples appeared to be between ~22 to 24 h, with the control having the greatest ESI of 23.9 h and the lowest of 22.5 h was observed for samples with the highest %DH. Simultaneously, a significant drop was observed in the concentration of adsorbed protein on the surface of oil droplets as it declined from 2.33 mg^−1^ mL^−1^ for the control to 1.50 mg^−1^ mL^−1^ for the actinidin-treated sample with 15% DH (Table 6) indicating a poorer surface coverage that likely resulted in diminished EAI. On the other hand, foam overrun and foam stability improved with the hydrolysis rate. Foam overrun increased from approximately 344% for the control to 406% for the sample with 15% DH. The foam stability of the sample was also improved, increasing from 1260 s to 2454 s (Table 6). Also, the protein patterns of the foam-drained liquid of MPC obtained during the foaming analysis are shown in Figure 4B and Table 4. In the MPC drained liquid, ~3% of αs- and β-CN, 5% of κ-CN, 9% of β-LG and 7% of α-LA remained at 15%DH.

#### 3.3.2. Functional Properties of WPC Hydrolysates Obtained by Actinidin

In the case of WPC (Figure 5), a similar trend to that of MPC was observed in relation to the solubility, which increased concomitantly with %DH. The untreated sample had about 83% solubility, which increased to about 88%, 93% and 97% at 5%, 10% and 15% DH, respectively (Figure 5). Proteolysis also significantly improved the heat stability, especially at its highest %DH. For example, from the control, heat stability increased from ~ 71% to the highest of ~95.1% at 15% DH (Figure 6). It can also be seen from Figure 3B and Table 3 that heat-treated hydrolysates had no visible bands after SDS-PAGE analysis of the WPC supernatant. However, at 0 and 5% DH, heat stability did not differ significantly (*p* > 0.05), and even at 10% DH, only about a 6% increase was observed, indicating that substantial hydrolysis was required to improve this functionality.

The EAI of the WPC control was 20.33 m^2^ g^−1^ and decreased to 17.45 m^2^ g^−1^ for the samples obtained after 15%DH. Also, there was a negligible change of EAI at 5% DH or only ~<1 of EAI change at 10% DH (Table 6). Furthermore, emulsion stability declined from 25.1 (control) to 24.5 h (hydrolysates with 15% DH). In contrast to MPC, WPC hydrolysates showed an increase in the concentration of adsorbed protein on the surface of oil droplets (Table 6) as the amount of proteins increased from 0.59 mg^−1^ mL^−1^ (control) to 0.80 mg^−1^ mL^−1^ (sample with highest DH).

Interestingly, the WPC control did not foam (overrun of 0%) under the experimental conditions, which was similar to the studies conducted by Dissanayake & Vasiljevic [28], reporting 0 s foam stability for whey proteins control sample, and Althouse, Dinakar, & Kilara, [29], where control whey protein isolate retentate showed no stable foam formation and 0% foam overrun. However, a great improvement in foaming of WPC was achieved to about 247, 252 and 270%, concomitant with an increase in %DH to 5, 10 and 15%, respectively. However, this increase in the foam overrun was accompanied by compromised foam stability. The most stable foam was the one with the lowest %DH (120 s), while the least stable foam was generated from the dispersion containing WPC with 15% DH (40 s). Similarly, the protein patterns of foam-drained liquid of WPC resulted in about 15% of β-LG and only about 5% of α-LA fractions remaining at 15% DH (Figure 4B and Table 4).

## 4. Discussion

Our previous studies showed that actinidin can be used to hydrolyse milk proteins to a certain extent, and the hydrolysates obtained had lower antigenicity in the case of both WPC and MPC substrates [10,11]. However, the use of actinidin for modulating the functional properties of dairy systems has not been assessed and applied. Therefore, the present study was carried out to explore the effect of actinidin hydrolysis on functional properties of commercial MPC and WPC, including solubility, heat stability, foaming and emulsification, as the latter two properties are also related to solubility [15].

Milk proteins have broad applicability in various food products due to their nutritional or physical properties. However, their application in food systems may be limited due to several important issues. For example, whey proteins are very soluble, a property highly dependent on the pH and/or temperature of the system, which creates problems during downstream processing and especially during manufacturing involving heat applications. Thus, partial hydrolysis may improve the stability of whey proteins by increasing their solubility and, thus, heat stability [15]. Also, milk protein concentrate usually has poor solubility, especially after prolonged storage [6], which may limit its functional properties. Thus, hydrolysis of milk proteins by proteases has the potential to address some of the issues leading to compromised functionalities [8]. Several studies have applied controlled enzymatic hydrolysis to enhance the functional properties of whey proteins, caseins and milk protein isolates. It has been observed that with a greater degree of hydrolysis, solubility can be increased with a concomitant decrease in viscosity [2,8,30].

Native WPs are globular with higher numbers of surface hydrophilic amino acid residues and buried hydrophobic and cysteine groups, resulting in high aqueous solubility [31,32]. In the case of MPC, poor solubility mainly occurs due to the structural rearrangement of the casein micelles that have a high hydrophobicity index [33]. Hydrophobic interactions, which take place between hydrophobic regions of caseins, are the main drivers of MPC insolubility [1,6,34].

Hydrolysis improved the solubility of both MPC and WPC further with the elevation of %DH (Figure 5), with whey proteins achieving almost full solubility at 15% DH. Even in MPC dispersions, about a 15% rise in solubility was observed at 15% DH in comparison to that of the control. Furthermore, the improvement in solubility can also be compared to the zeta potential of MPH in the case of both substrates. Hydrolysates were characterised by a greater net-negative zeta potential compared to that of the controls, where both bulk and supernatant of both substrates mostly resulted in greater negative zeta potential and, thereby, higher solubility through likely enhanced electrostatic repulsions. The changes in solubility were also reflected in the electrophoretic patterns of both substrates. α_s_-CN in the case of caseins and α-LA in the case of whey proteins were mostly affected fractions, which resulted in hydrolysates with smaller molecular weight oligopeptides with an increase in %DH, as observed in the SDS-PAGE gels, which consequently resulted in improved solubility. However, while hydrolysis improved solubility, which can also be related to reduced average particle size, the extent of proteolysis appears to be also relevant as the greater DH, i.e., 15% (MPC bulk), resulted in a substantial rise in the average particle size (up to 343 nm from 295 nm) likely indicating aggregation. However, this aggregation was not visible in the gels. This can be due to the nature of these aggregates, as they could have been created via weak forces easily broken by SDS. During proteolysis, cleavage of peptide bonds takes, which causes a release of the number of amino and carboxyl groups, resulting in an increment in hydrophilicity and net charge density of the hydrolysates obtained by promoting proteins-water interactions [35].

According to previous studies, β-sheet and α helix contents of native and unhydrolysed β-lactoglobulin comprise about 43–50% and 10–15% of all structural elements, respectively [36]. On the other hand, α-LA has about 18% and 36% [36], α_s2_-CN about 27% and 32% [37], β-CN about 34% and 29% [37], κ-CN has about 39–41% and 8–10% of these structural elements [38], respectively. Furthermore, αs_1_-CN has only a small amount of secondary structure containing only α-helices or β-sheets. In the current study, despite negligible change in the contents of β-turn and random coils, in the case of both substrates at maximum DH, a significant change was seen in the content of β-sheet (increased) and α-helical (declined) in comparison to the unhydrolysed samples. This implies that the actinidin hydrolysis may have resulted in conformational rearrangements, transforming these proteins from a predominant α-helical to a β-sheet form. Both β-sheet and α-helical structures are mainly created by hydrogen bonds between amine hydrogen and carbonyl oxygen atoms that construct the peptide backbone of the substrates [36]. The FTIR analysis (Table 5) showed that the protein structure was changed substantially, which likely led to the exposure of previously buried hydrophilic regions to the environment [15] and consequently improved solubility. It can also be seen in Supplementary Materials Figure S1, where spectra of WPC hydrolysates containing 15% DH showed the highest peaks.

Furthermore, significant increases in heat stability for whey proteins after hydrolysis are in agreement with a study conducted by Castro & Sato [39], in which high solubility and high heat stability were recorded after hydrolysis of whey proteins with Flavourzyme^®^. For WPC, it is critical to have appropriate heat stability as these proteins usually go through reconstitution and heat treatment during manufacturing, which may cause end-product destabilisation. In the case of MPC, heat instability occurs regardless of the fact that caseins can withstand higher temperature treatment without aggregation. Heat instability is also caused by whey proteins (mostly driven by β-LG due to its higher concentration in whey) denaturation and their reaction with casein micelles [40]. κ-CN and β-LG complex (colloidal or serum) are associated with regions of maximum and minimum heat stability, respectively [41,42]. Various studies have been conducted on milk proteins with the main focus on their heat stability [1,3,43]. Also, a study conducted by Gauthier & Pouliot [44] showed improved heat stability of hydrolysed whey proteins in an acidic beverage.

While notable improvement in solubility and heat stability has been observed upon substrate hydrolysis using actinidin, both substrates experienced a reduction in EAI and ESI. A similar trend has been reported by Slattery & Fitzgerald [45] when sodium caseinate hydrolysates were obtained by *Bacillus* proteinase and another study by Singh & Dalgleish [46] when commercial range of whey protein hydrolysates was tested for emulsifying properties. Emulsifying properties mainly depend on both surface hydrophobicity and molecular flexibility of proteins [47]. The greater emulsifying activity index appears with improvement in interfacial properties due to partial unfolding of proteins [48]. A greater amount of adsorbed proteins was present in the case of whey proteins as DH increased. However, greater hydrolysis of MPC resulted in a lesser amount of adsorbed proteins on the surface of fat droplets, which led to poorer emulsification. Reduction in emulsification activity occurred either due to the presence of a greater amount of hydrophilic peptides that lack or limit binding to the oil–water interface or the absence of a strong interfacial layer to prevent recoalescence of the oil [15]. It is well known that α-LA has poor gelling but good emulsifying properties, whereas β-LG exhibits excellent gelling, foaming and emulsifying properties [25]. In the current study, the progressive disappearance of α-LA bands in Figure 2D and Figure 3B demonstrate preferential hydrolysis of this protein fraction to smaller peptides, hence likely reduction of EAI and their stability. Also, emulsion stability was slightly decreased in the case of both substrates compared to their controls. Lower ESI may be due to a rise in the number of polar groups resulting from proteolysis, which altered a protein structure and thus enhanced hydrophilicity. A similar trend has been reported previously by Severin & Xia [15] and Singh & Dalgleish [46] when they used whey protein hydrolysates, and Slattery & Fitzgerald [45] used sodium caseinate hydrolysates created by different proteases.

According to Damodaran [9], partial hydrolysis of milk proteins generally improves foaming properties, whereas extensive hydrolysis can adversely affect it. In the current study, improvement in foam stability (WPC only at 5% DH) and overrun (MPC and WPC at all levels of DH) was observed. Foam stability is extensively dependent on the rheological as well as adhesive properties of interfacial film. Poor foam stability can occur due to capillary drainage of lamellae and rapid collapsing of bubbles [29]. The high foam stability at 5% DH can be attributed to the larger molecular weight of released peptides that directly influence foam stability compared to hydrolysates with higher DH and smaller peptides and free amino acids. Similarly, Althouse et al. [29] prepared foams with 5% whey hydrolysate at pH 7 that showed improved foam capacity (percent overrun). The good foamability of MPC can be attributed to the high flexibility of the casein structure, with similar results being reported by many studies [49,50,51]. Proteolysis leads to an increase of polypeptide and amino acids content of hydrolysates that enhances the incorporation of air at the air-water interface and thus improves foam capacity [52].

## 5. Conclusions

Actinidin hydrolysis of MPC and WPC resulted in improvement in certain functional properties. Protein solubility of both substrates increased with increasing DH, with the highest solubility achieved at 15% DH, where especially WPC showed almost full solubility (~97%). Heat stability also increased in the case of WPC only; however, the mixed trend was seen in the case of MPC with maximum heat stability at 5% DH. Despite improvement in solubility and heat stability, both hydrolysed substrates showed poor emulsifying properties compared to these of the intact proteins, with MPC at 15% DH having the lowest EAI among all substrates. Actinidin-induced hydrolysis also improved foaming properties for both substrates, including foaming stability, where MPC with 15% DH had the greatest foam stability of almost double that of the control. The only exception was hydrolysates of whey proteins at 15% DH with a highly compromised foam stability. These results indicate that actinidin can be used to solubilise MPs, thereby improving the functionality of milk proteins (such as solubility, heat stability, foaming stability and overrun) in different foods.

## Figures and Tables

**Table 1:** Average particle diameter and zeta potential of the whole samples and supernatants of milk protein hydrolysates (MPH) obtained from milk protein concentrate (MPC) and whey protein concentrate (WPC) incubated with actinidin to a degree of hydrolysis (%DH) of 0, 5, 10 or 15 at 60 °C.

MPH (%DH)	Particle Diameter (nm)				Zeta Potential (mV)			
	MPC		WPC		MPC		WPC	
	MPC Bulk	MPC Supernatant	WPC Bulk	WPC Supernatant	MPC Bulk	MPC Supernatant	WPC Bulk	WPC Supernatant
0	295 ± 4 ^b^	282 ± 4 ^a^	426 ± 1 ^a^	386 ± 3 ^a^	−2 ± 0.3 ^a^	−15 ± 0.0 ^ab^	−9 ± 0.0 ^a^	−9 ± 0.4 ^a^
5	281 ± 2 ^d^	233 ± 12 ^b^	419 ± 3 ^b^	381 ± 1 ^b^	−4 ± 0.6 ^b^	−16 ± 1.3 ^b^	−10 ± 0.4 ^b^	−10 ± 0.4 ^b^
10	290 ± 3 ^c^	180 ± 8 ^c^	409 ± 4 ^c^	365 ± 1 ^c^	−7 ± 0.0 ^c^	−14 ± 0.2 ^a^	−10 ± 1.9 ^b^	−12 ± 2.2 ^c^
15	343 ± 5 ^a^	171 ± 5 ^d^	410 ± 3 ^c^	353 ± 3 ^d^	−3 ± 1.9 ^ab^	−18 ± 0.0 ^c^	−13 ± 0.4 ^c^	−14 ± 0.2 ^d^

The values are presented as means of subsampling of three independent observations plus or minus standard deviation (SD). The values with different lower-case letters indicate significant differences (*p* < 0.05) within a column.

**Table 2:** Residual intact milk proteins in the whole samples and supernatant of dispersions of milk protein concentrate (MPC) and whey protein concentrate (WPC) incubated with actinidin to a degree of hydrolysis (%DH) of 0, 5, 10 and 15 at 60 °C.

Protein	Proportion of Proteins Remaining Relative to Control (%)					
	DH (%)					
	5	10	15	5	10	15
**MPC**	**Solubility**					
	**Whole Sample**			**Supernatant**		
α_s_-CN	54.6 ± 3.2 ^aE^	27.4 ± 2.1 ^cD^	8.1 ± 0.5 ^eD^	38.7 ± 3.4 ^bC^	14.8 ± 0.9 ^dB^	3.0 ± 0.2 ^fB^
β-CN	57.4 ± 0.0 ^aD^	30.5 ± 0.0 ^bC^	7.0 ± 0.0 ^eE^	27.0 ± 0.0 ^cE^	19.2 ± 0.0 ^dA^	4.6 ± 3.6 ^fA^
κ-CN	43.7 ± 0.1 ^aG^	19.8 ± 0.0 ^bE^	5.9 ± 0.0 ^dF^	20.2 ± 0.0 ^bG^	7.0 ± 0.0 ^cD^	0.0 ± 0.0 ^eD^
β-LG	61.5 ± 3.6 ^bC^	31.9 ± 3.3 ^cC^	9.6 ± 1.0 ^eC^	63.7 ± 3.5 ^aA^	13.4 ± 0.0 ^dC^	2.0 ± 0.0 ^fC^
α-LA	46.0 ± 4.1 ^aF^	18.3 ± 1.7 ^cF^	0.0 ± 0.0 ^dG^	43.0 ± 1.3 ^bB^	0.0 ± 0.0 ^dE^	0.0 ± 0.0 ^dD^
**WPC**	**Whole sample**			**Supernatant**		
β-LG	81.2 ± 3.9 ^aB^	60.6 ± 1.7 ^bA^	25.5 ± 0.5 ^dA^	36.8 ± 0.5 ^cD^	19.0 ± 0.0 ^eA^	0.0 ± 0.0 ^fD^
α-LA	83.5 ± 1.6 ^aA^	45.7 ± 2.0 ^bB^	21.5 ± 0.5 ^dB^	23.9 ± 0.6 ^cF^	0.0 ± 0.0 ^eE^	0.0 ± 0.0 ^eD^

The values are presented as means of at least three independent observations ± standard deviation (SD); lower and upper-case superscript letters indicate significant differences (*p* < 0.05) within a row and a column, respectively.

**Table 3:** Proportion (%) of milk proteins remaining (relative to control) after heat stability of varying degrees of hydrolysis of MPC or WPC by actinidin at 60 °C. Where (-) is not determined.

Proportion of Proteins Remaining Relative to Control (%) during Heat Stability						
DH (%)	5	10	15	5	10	15
Protein	Supernatant (MPC)			Supernatant (WPC)		
α_s_-CN	28.2 ± 2.1 ^aD^	13.4 ± 0.4 ^bC^	10.8 ± 0.3 ^cC^	-	-	-
β-CN	32.3 ± 1.8 ^aC^	21.0 ± 1.6 ^bB^	20.0 ± 1.7 ^cB^	-	-	-
κ-CN	28.0 ± 1.3 ^aD^	11.1 ± 0.9 ^bD^	9.7 ± 0.9 ^cD^	-	-	-
β-LG	51.7 ± 2.6 ^aA^	32.3 ± 2.2 ^cA^	25.7 ± 2.3 ^dA^	48.5 ± 3.1 ^bA^	22.3 ± 2.5 ^eA^	0.0 ± 0.0 ^fA^
α-LA	38.0 ± 2.8 ^bB^	0.0 ± 0.0 ^dE^	0.0 ± 0.0 ^dE^	47.7 ± 2.1 ^aB^	11.3 ± 4.5 ^cB^	0.0 ± 0.0 ^dA^

The values are means of at least three independent observations ± standard deviation (SD); lower and upper-case superscript letters indicate significant differences (*p* < 0.05) within a row and a column, respectively.

**Table 4:** Proportion (%) of milk proteins remaining (relative to unhydrolysed control) after foaming. Where (-) is not applicable.

Proportion of Proteins Remaining Relative to Control (%) during Foaming						
DH (%)	5	10	15	5	10	15
Protein	Foam Drained Liquid (MPC)			Foam Drained Liquid (WPC)		
α_s_-CN	18.0 ± 1.3 ^aE^	7.8 ± 0.3 ^bD^	3.4 ± 0.2 ^cD^	-	-	-
β-CN	27.1 ± 0.5 ^aC^	13.8 ± 0.5 ^bB^	2.8 ± 0.0 ^cE^	-	-	-
κ-CN	26.3 ± 1.2 ^aD^	11.3 ± 0.0 ^bC^	5.0 ± 0.3 ^cC^	-	-	-
β-LG	33.2 ± 2.4 ^aB^	15.1 ± 0.0 ^cA^	9.4 ± 0.0 ^dA^	33.8 ± 0.7 ^aA^	18.7 ± 1.5 ^bB^	15.4 ± 0.6 ^cA^
α-LA	36.8 ± 0.3 ^aA^	11.6 ± 0.0 ^dC^	7.3 ± 0.0 ^eB^	26.4 ± 2.4 ^bB^	25.7 ± 2.3 ^cA^	4.7 ± 0.4 ^fB^

Values are means of at least three independent observations ± standard deviation (SD); upper-case and lower-case superscript letters indicate significant differences (*p* < 0.05) within a column and a row, respectively.

**Table 5:** Proportion of defined structural elements of milk proteins observed within broad amide I region (1700–1600 cm^−1^) measured by FTIR after hydrolysis (MPH) of milk protein concentrate (MPC) and whey protein concentrate (WPC) by actinidin to 0, 5, 10 and 15% DH at 60 °C.

MPH	Band Assignment	Degree of Hydrolysis (%)							
		Control (0)		5		10		15	
		Band Frequency (cm^−2^)	Peak Area %	Band Frequency (cm^−2^)	Peak Area %	Band Frequency (cm^−2^)	Peak Area %	Band Frequency (cm^−2^)	Peak Area %
MPC	β-sheet	1638–1608, 1691–1681	42.4 ± 8.4 ^dB^	1640–1608, 1693–1681	45.2 ± 1.2 ^cB^	1640–1609, 1693–1681	47.7 ± 3.1 ^bB^	1640–1610, 1693–1682	53.2 ± 1.0 ^aA^
	Random coil	1647–1643	10.6 ± 1.9 ^bG^	1648–1643	11.6 ± 4.3 ^aG^	1646–1642	8.4 ± 1.4 ^cG^	1646–1642	8.2 ± 0.4 ^cD^
	α-helix	1662–1651	22.1 ± 3.3 ^aC^	1662–1650	22.5 ± 1.4 ^aC^	1662–1650	19.8 ± 1.6 ^bC^	1662–1650	15.9 ± 0.8 ^cB^
	β-turn	1677–1666	17.4 ± 1.2 ^bE^	1678–1667	17.6 ± 5.3 ^bE^	1678–1667	18.8 ± 1.4 ^aD^	1677–1666	16.1 ± 1.4 ^cB^
WPC	β-sheet	1639–1610, 1691–1681	49.6 ± 1.3 ^bA^	1639–1610, 1691–1680	49.6 ± 0.8 ^bA^	1640–1610, 1690–1681	53.3 ± 5.0 ^aA^	1640–1610, 1691–1681	53.4 ± 1.5 ^aA^
	Random coil	1648–1643	9.0 ± 0.7 ^bH^	1647–1643	11.2 ± 0.4 ^aG^	1646–1642	8.7 ± 1.8 ^bG^	1646–1642	9.0 ± 0.3 ^bC^
	α-helix	1663–1650	20.6 ± 1.3 ^aD^	1663–1650	18.3 ± 1.5 ^bD^	1661–1649	14.8 ± 3.2 ^dE^	1660–1649	15.9 ± 1.1 ^cB^
	β-turn	1677–1666	15.5 ± 1.4 ^bF^	1678–1667	16.8 ± 1.2 ^aF^	1677–1666	11.7 ± 2.4 ^cF^	1678–1666	15.8 ± 2.0 ^bB^

Values are means of at least three independent observations ± standard deviation (SD); The lower-case superscript letters indicate significant differences (*p* < 0.05) within a row, and the upper-case letters indicate significant differences (*p* < 0.05) within a column.

**Table 6:** Adsorbed proteins, emulsifying and foaming properties of milk protein hydrolysates (MPH) obtained from milk protein concentrate (MPC) and whey protein concentrate (WPC) by actinidin to 0, 5, 10 and 15% DH at 60 °C.

MPH (%DH)	Foam Stability (s)	Overrun (%)	*EAI* (m^2^ g^−1^)	Adsorbed Protein (mg^−1^ mL^−1^)	*ESI* (h)
**Hydrolysates from MPC**					
0	1260 ± 8 ^d^	344.8 ± 4 ^c^	17.45 ± 0.0 ^c^	2.33 ± 0.2 ^a^	23.9 ± 0.1 ^d^
5	1904 ± 10 ^c^	349.7 ± 3 ^c^	13.80 ± 0.4 ^d^	1.72 ± 0.1 ^b^	23.8 ± 0.1 ^d^
10	2160 ± 6 ^b^	358.5 ± 2 ^b^	11.58 ± 0.8 ^e^	1.56 ± 0.1 ^bc^	22.9 ± 0.2 ^e^
15	2454 ± 2 ^a^	406 ± 3 ^a^	9.49 ± 0.1 ^f^	1.50 ± 0.2 ^c^	22.5 ± 0.5 ^f^
**Hydrolysates from WPC**					
0	7 ± 1 ^h^	0 ± 0.0 ^f^	20.33 ± 0.3 ^a^	0.59 ± 0.1 ^f^	25.1 ± 0.5 ^a^
5	120 ± 2 ^e^	247.6 ± 4 ^e^	20.24 ± 0.9 ^a^	0.73 ± 0.1 ^de^	24.8 ± 0.7 ^b^
10	105 ± 6 ^f^	252.1 ± 4 ^e^	19.22 ± 0.7 ^b^	0.77 ± 0.1 ^d^	24.6 ± 0.4 ^bc^
15	40 ± 4 ^g^	270.4 ± 3 ^d^	17.45 ± 0.2 ^c^	0.80 ± 0.0 ^d^	24.5 ± 0.3 ^bc^

The values are the mean of at least three independent observations ± standard deviation (SD); lower-case superscript letters indicate significant differences (*p* < 0.05) within a column.

## Data Availability

The data is contained within the document.

## Supplementary Materials

The following supporting information can be downloaded at https://www.mdpi.com/article/10.3390/foods12203806/s1, Figure S1: Second derivative of amide I region (1700–1600 cm^−1^) of MPH of milk protein concentrate (MPC) and whey protein concentrate (WPC) by actinidin to 0, 5, 10 and 15% DH at 60 °C.
