# Supplementary material for: Influence of Actinidin-Induced Hydrolysis on the Functional Properties of Milk Protein and Whey Protein Concentrates
Source: Foods. 2023 Oct 17;12(20):3806. doi: 10.3390/foods12203806 (PMC10606088; doi:10.3390/foods12203806)

S1: Second derivative of amide I region (1700–1600 cm^{-1}) of MPH of milk protein concentrate (MPC, A) and whey protein concentrate (WPC, B) by actinidin to 0, 5, 10 and 15% DH at 60°C.

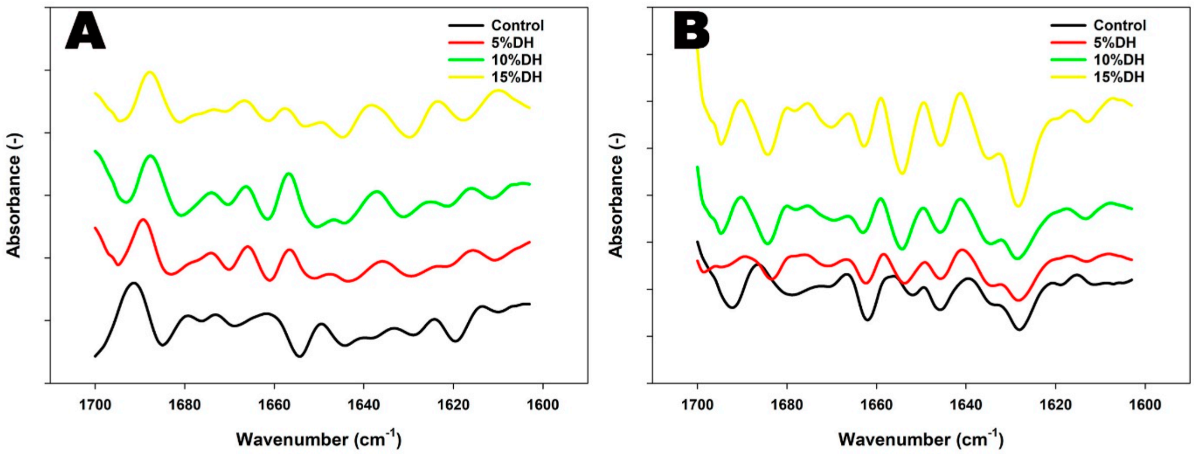

Supplement: Supplementary file 1 [file foods-12-03806-s001.zip › foods-2654085-supplementary.pdf]
